# Zinc depletion regulates the processing and secretion of IL-1*β*

**DOI:** 10.1038/cddis.2013.547

**Published:** 2014-01-30

**Authors:** H Summersgill, H England, G Lopez-Castejon, C B Lawrence, N M Luheshi, J Pahle, P Mendes, D Brough

**Affiliations:** 1 Faculty of Life Sciences, University of Manchester, AV Hill Building, Oxford Road, Manchester, UK; 2 MedImmune Ltd, Aaron Klug Building, Granta Park, Cambridge, UK; 3 School of Computer Science, University of Manchester, Manchester, UK

**Keywords:** inflammation, caspase-1, interleukin-1, inflammasome, zinc

## Abstract

Sterile inflammation contributes to many common and serious human diseases. The pro-inflammatory cytokine interleukin-1*β* (IL-1*β*) drives sterile inflammatory responses and is thus a very attractive therapeutic target. Activation of IL-1*β* in sterile diseases commonly requires an intracellular multi-protein complex called the NLRP3 (NACHT, LRR, and PYD domains-containing protein 3) inflammasome. A number of disease-associated danger molecules are known to activate the NLRP3 inflammasome. We show here that depletion of zinc from macrophages, a paradigm for zinc deficiency, also activates the NLRP3 inflammasome and induces IL-1*β* secretion. Our data suggest that zinc depletion damages the integrity of lysosomes and that this event is important for NLRP3 activation. These data provide new mechanistic insight to how zinc deficiency contributes to inflammation and further unravel the mechanisms of NLRP3 inflammasome activation.

Inflammation is a protective host response required for resistance to infection. However, inflammation that occurs in response to tissue injury in the absence of pathogen is considered sterile, and can contribute to damage.^1^ Sterile inflammation is driven by the pro-inflammatory cytokines of the interleukin-1 (IL-1) family.^1^ IL-1*β* is a master cytokine central to the damaging inflammatory response in a range of major human diseases.^2^ For this reason, understanding the mechanisms of IL-1*β* production is a crucial area of research that may lead to the identification of new therapeutic targets and therapies. IL-1*β* is produced by macrophages as a 31-kDa precursor called pro-IL-1*β*. Pro-IL-1*β* is expressed in response to pathogen-associated molecular patterns or damage-associated molecular patterns (DAMPs) that bind to pattern recognition receptors (PRRs) on the macrophage to upregulate pro-inflammatory gene expression.^3, 4^ Pro-IL-1*β* is inactive and remains intracellular until a further pathogen-associated molecular pattern or DAMP stimulation activates cytosolic PRRs, often of the NOD-like receptor family, to form large multi-protein complexes called inflammasomes.^5^ These complexes consist of the PRR, pro-caspase-1, and depending upon the PRR, an adaptor protein called ASC (apoptosis-associated speck-like protein containing a caspase recruitment domain), that interact via homotypic interactions between caspase activation and recruitment and pyrin (PYD) domains.^5^ Of the inflammasomes identified to-date, the best characterised and most relevant to sterile inflammatory responses is formed by the PPR NLRP3 (NACHT, LRR, and PYD domains-containing protein 3).^5, 6^ When NLRP3 senses DAMPs it recruits ASC, which in turn recruits caspase-1 causing its activation. Caspase-1 then processes pro-IL-1*β* to a mature form that is rapidly secreted from the cell.^5^

Zinc (Zn^2+^) is an essential nutrient, serving both structural and catalytic cofactor roles for many proteins (up to 10% of the human proteome binds Zn^2+^^7^). Zn^2+^ is also central to many key molecular and cellular reactions critical for innate and adaptive immune system function.^8^ Even though total levels of cellular Zn^2+^ are high, the levels of available Zn^2+^ are tightly regulated and the level of available Zn^2+^ in plasma drops rapidly in response to bacterial endotoxin or cytokines.^9, 10^ Zn^2+^ deficiency is common in older people^11^ and contributes to many clinical disorders, such as growth retardation, immune dysfunction, and cognitive impairment, affecting up to 2 billion people worldwide.^12^ Zn^2+^ supplementation is reported to be beneficial at ameliorating the effects of these disorders and in many infectious and non-infectious diseases.^13, 14^

We reported previously an interaction between Zn^2+^ and caspase-1-dependent processing and release of IL-1*β*.^15^ A brief 15 min incubation of lipopolysacharide (LPS)-primed peritoneal macrophages with the Zn^2+^ chelator TPEN (*N,N,N',N'*-Tetrakis-(2–pyridylmethyl) ethylenediamine) inhibits IL-1*β* release in response to NLRP3 inflammasome agonists ATP and nigericin.^15^ However, here we have discovered that sustained Zn^2+^ depletion acts as a stimulus for the NLRP3 inflammasome. These data provide valuable insights into regulation of the NLRP3 inflammasome, and the mechanisms through which Zn^2+^ deficiency may contribute to inflammatory disease.

## Results and Discussion

**Figure 1:**
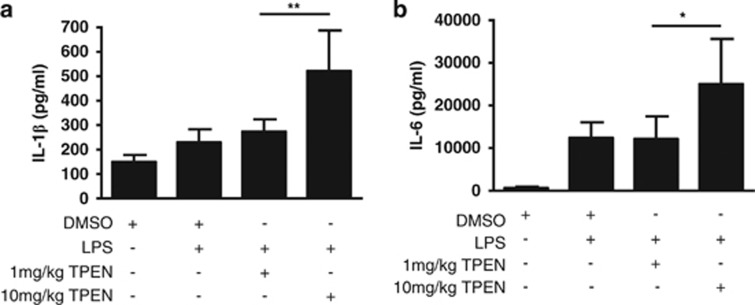
TPEN is pro-inflammatory *in vivo.* Male C57BL/6 mice were injected i.p. with a septic dose (5 mg/kg) of LPS. 1 h after LPS injection the mice received a second injection that was vehicle (10% DMSO in saline), 1 mg/kg TPEN, or 10 mg/kg TPEN. 3 h after the second injection the animals were anaesthetised, lavage fluid was collected, and analysed for the presence of IL-1*β* (**a**) or IL-6 (**b**) by specific ELISA. Data were collected from six animals per group and are presented as the mean±S.D. **P<0.01, *P<0.05

We have previously reported that brief (15 min) incubation of LPS-primed mouse peritoneal macrophages with TPEN completely inhibits ATP- and nigericin-induced caspase-1 activation and secretion of IL-1*β*.^15^ We discovered that this was probably due to an inhibition of the pannexin-1 hemichannel. To test whether these data are relevant to inflammation *in vivo,* adult C57BL/6 mice were injected intraperitoneally (i.p.) with LPS (5 mg/kg) followed by TPEN (1 or 10 mg/kg) 1 h later, with peritoneal lavages recovered 3 h following TPEN administration. Levels of IL-1*β* and of another pro-inflammatory cytokine, IL-6, in the lavage fluid were analysed by ELISA. In contrast to our previous *in vitro* data,^15^ TPEN *in vivo* was pro-inflammatory, increasing the levels of both IL-1*β* and IL-6 (Figures 1a and b).

**Figure 2:**
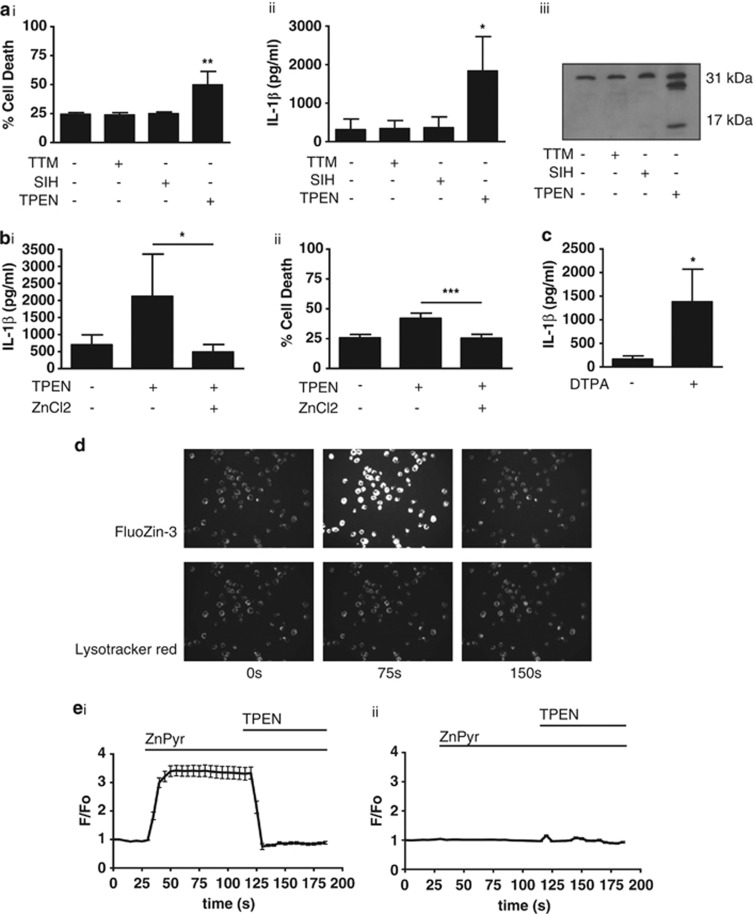
Zn^2+^ depletion drives processing and secretion of IL-1*β* from macrophages. (**a**) Mouse primary peritoneal macrophages were primed with LPS (1 *μ*g/ml, 2 h), followed by 4 h treatment with 10 *μ*M TPEN, the copper chelator TTM, or the iron chelator SIH with effects on cell death measured by LDH release (i), IL-1*β* release measured by ELISA (ii), and IL-1*β* processing measured by western blot analysis (iii). The band at 31 kDa is pro-IL-1*β* and the band at 17 kDa is IL-1*β*. (**b**) Mouse primary peritoneal macrophages were primed with LPS (1 *μ*g/ml, 2 h), followed by treatment with TPEN (10 *μ*M, 4 h)±50 *μ*M ZnCl_2_ with release of IL-1*β* measured by ELISA (i) and cell death by LDH release (ii). (**c**) Mouse primary peritoneal macrophages were primed with LPS (1 *μ*g/ml, 2 h), followed by 4 h treatment with the non-cell permeable Zn^2+^ chelator DTPA (1 mM) with release of IL-1*β* measured by ELISA. (**d**) J774 macrophages were loaded with the Zn^2+^ indicator FluoZin-3 and Lysotracker Red and then treated with 1 *μ*M of the Zn^2+^ ionophore, 1-hydroxypyridine-2-thione (zinc salt) (ZnPyr), to induce increases in cellular Zn^2+^. Addition of TPEN to a Zn^2+^-loaded cell caused a rapid drop in FluoZin-3 fluorescence. Shown are snap shot images taken with a BD Pathway illustrating the fluorescence changes over the course of the experiment. Scale bar=50 *μ*m. (**e**) Representative fluorescent traces (F/Fo) of FluoZin-3 (i) and lysotracker red (ii) of the experiment shown in **d**. All data are presented as the mean±S.D. from at least four separate experiments. Blots shown are representative. ***P<0.001, **P<0.01, *P<0.05

We therefore asked the question whether, in contrast to our report on the effects of short-term TPEN treatment on NLRP3 inflammasome activation,^15^ sustained Zn^2+^ depletion with TPEN could activate IL-1*β* processing and release. To test whether sustained (4 h) Zn^2+^ depletion modifies the production and secretion of IL-1*β* directly, we treated cultured mouse primary peritoneal macrophages with TPEN (0–10 *μ*M) or dimethyl sulfoxide (DMSO; 0–0.5%) for 4 h and measured the levels of IL-1*β* in cell lysates and culture supernatants by ELISA. Under these conditions TPEN did not induce significant expression or release of IL-1*β* (data not shown). After priming cultured macrophages with LPS (1 *μ*g/ml, 2 h), TPEN treatment (10 *μ*M, 4 h) induced the release of mature (17 kDa) IL-1*β* (Figure 2aii, iii), and caused cell death (Figure 2ai). This is consistent with models of NLRP3-inflammasome-dependent IL-1*β* secretion where an initial priming stimulus is required to induce the expression of pro-IL-1*β* and the PRR NLRP3.^16^ The effect of TPEN on cell death and IL-1*β* processing and release was specific to a depletion of Zn^2+^ as 4 h incubation with selective copper (TTM, ammonium tetrathiomolybdate) or iron (SIH, salicylaldehyde isonicotinoyl hydrazone) chelators had no effect on either parameter (Figure 2a). The addition of ZnCl_2_ (50 *μ*M) to TPEN-treated LPS-primed macrophage cultures reduced cell death and inhibited IL-1*β* release (Figure 2b). Another Zn^2+^ chelator (DTPA, diethylenetriaminepentaacetic acid^17^) also induced the release of IL-1*β* (Figure 2c). We confirmed that the addition of TPEN quenched cellular Zn^2+^ using the following protocol. LPS-primed J774 macrophages were loaded with the selective Zn^2+^ indicator FluoZin-3 and a fluorescent lysosomal marker, Lysotracker Red, and were imaged using a BD Pathway Bioimager (San Jose, CA, USA). Addition of 1 *μ*M of the Zn^2+^ ionophore, 1-hydroxypyridine-2-thione (zinc salt; ZnPyr) rapidly increased the FlouZin-3 fluorescence in the cells saturating the dye (<20 s), but had no effect on lysotracker red fluorescence (Figures 2d and e). Addition of TPEN to a Zn^2+^-loaded cell caused a rapid drop in FluoZin-3 fluorescence confirming the specificity of the response (Figures 2d and e). Thus, sustained depletion of Zn^2+^ was toxic and induced the processing and release of IL-1*β* analogous to other known danger signals. The effects of Zn^2+^ depletion on IL-1*β* release described here may help to explain, at least in part, the well-documented contribution of Zn^2+^ deficiency to inflammation and disease.

**Figure 3:**
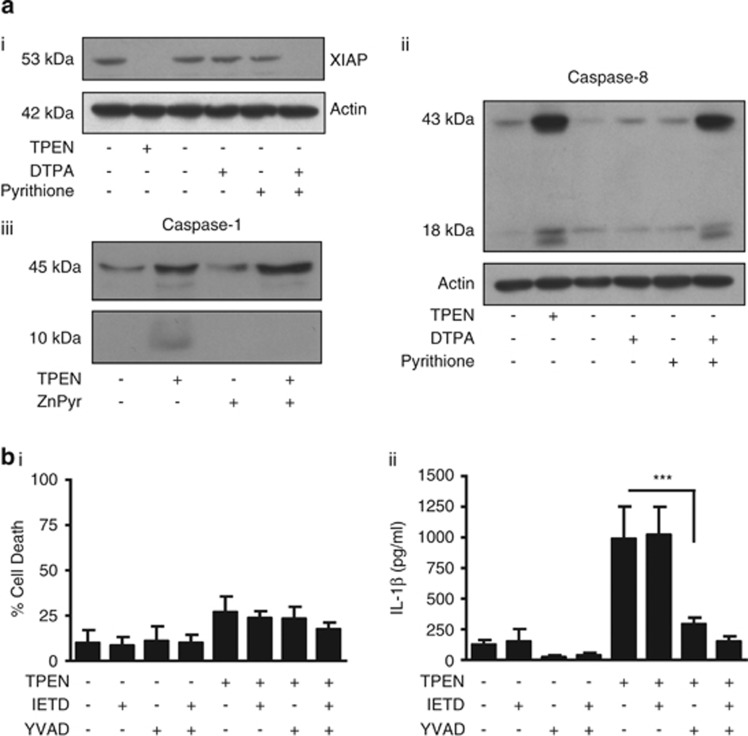
TPEN-induced IL-1*β* release is dependent upon caspase-1. (**a**) Lysates of mouse peritoneal macrophages treated with LPS (1 *μ*g/ml, 2 h) and TPEN (10 *μ*M, 4 h), or DTPA (1 mM) and pyrithione (50 *μ*M), or TPEN±1 *μ*M of 1-hydroxypyridine-2-thione (zinc salt) (ZnPyr), were blotted for the house keeping protein actin (band at 42 kDa) and for XIAP (band at 53 kDa) (i). Lysates were also blotted for active caspase-8 sub-units (43 and 18 kDa) (ii), and for pro- (45 kDa) and active (10 kDa) caspase-1 (iii). (**b**) LPS-primed primary peritoneal macrophages were treated with TPEN (10 *μ*M, 4 h) plus and minus incubation with the caspase-1 inhibitor Ac-YVAD-CHO (100 *μ*M) or the caspase-8 inhibitor IETD-CHO (100 *μ*M) with cell death measured by LDH assay (i) and IL-1*β* release by ELISA (ii). All data are presented as the mean±S.D. from at least four separate experiments. Blots shown are representative. ***P<0.001

To investigate the mechanism through which TPEN induced IL-1*β* release, we initially investigated whether release was dependent upon caspase-1 or caspase-8. TPEN induces tumour cell death by causing the degradation of X-linked inhibitor of apoptosis protein (XIAP),^18, 19^ and inhibitor of apoptosis protein inhibitors induce IL-1*β* processing via both NLRP3/caspase-1 and caspase-8-dependent mechanisms.^20^ Once activated caspase-8 can cleave pro-IL-1*β* directly at the same site as caspase-1, and induce secretion of the mature form.^20, 21^ Cell death can also induce the release of IL-1*β* from macrophages that is dependent upon caspase-8, but is independent of inflammasomes.^22^ Thus, we investigated whether TPEN-induced IL-1*β* processing and secretion were dependent on caspase-1 or caspase-8. Treatment of LPS-primed primary peritoneal macrophages with TPEN resulted in the loss of XIAP and an activation of caspase-8 (Figure 3ai). The extracellular Zn^2+^ chelator DTPA and the Zn^2+^ ionophore pyrithione also induced a loss of XIAP and an activation of caspase-8 (Figure 3ai, ii). TPEN treatment also induced activation of caspase-1, as seen by the appearance of the active caspase-1 subunit (10 kDa) in TPEN-treated culture supernatants (Figure 3aiii). This activation of caspase-1 was inhibited by the addition of 1 *μ*M ZnPyr confirming the Zn^2+^ dependence of this effect. Thus, from these data it is clear that Zn^2+^ depletion activated both caspase-1 and caspase-8. To determine which of these caspases were involved in TPEN-induced IL-1*β* processing and release, we used selective caspase-1 and caspase-8 inhibitors. The caspase-1 inhibitor Ac-YVAD-CHO or the caspase-8 inhibitor IETD-CHO had no effect on TPEN-induced cell death (Figure 3bi). However, although IETD had no effect on TPEN-induced IL-1*β* secretion (Figure 3bii), incubation of TPEN-treated macrophages with YVAD resulted in inhibition of IL-1*β* release, suggesting that TPEN-induced IL-1*β* processing and release were caspase-1 dependent (Figure 3bii). Thus, TPEN induced the activation of caspase-1, and the processing and release of IL-1*β*, which was blocked by a selective caspase-1 inhibitor.

**Figure 4:**
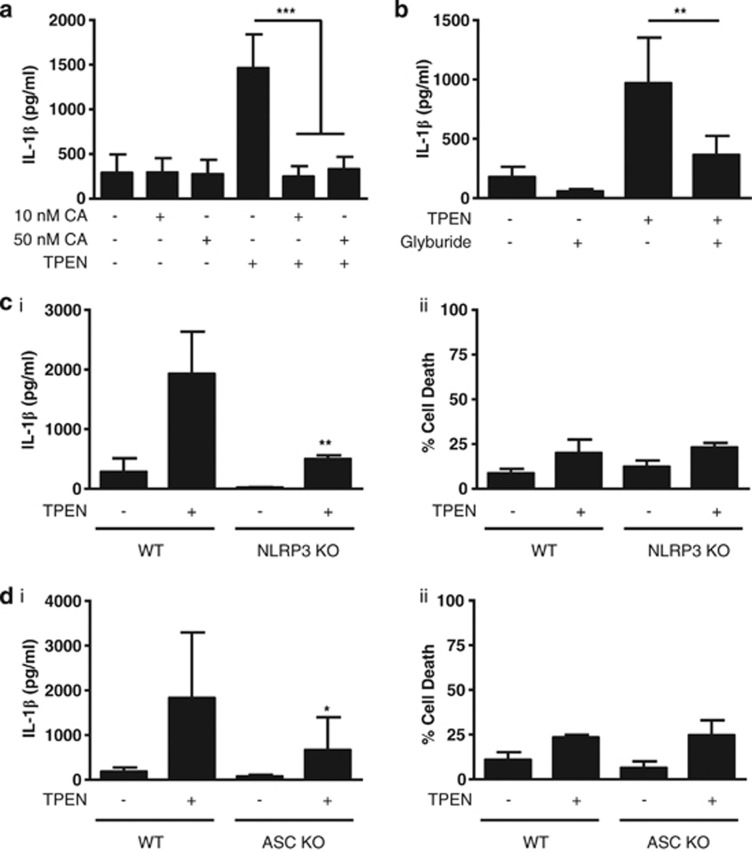
TPEN-induced IL-1*β* release is dependent upon the NLRP3 inflammasome. (**a**) LPS-primed primary peritoneal macrophages were incubated with CA (10 or 50 nM) followed by TPEN (10 *μ*M, 4 h) with IL-1*β* release measured by ELISA. (**b**) LPS-primed peritoneal macrophages were incubated with the NLRP3 inflammasome inhibitor glyburide (100 *μ*M) before incubation with TPEN (10 *μ*M, 4 h) with IL-1*β* release measured by ELISA. (**c**) Macrophages isolated from WT and NLRP3 KO mice were LPS-primed (1 *μ*g/ml, 2 h) and then treated with TPEN (10 *μ*M, 4 h) with IL-1*β* release measured by ELISA (i), and cell death by LDH release (ii). (**d**) Macrophages isolated from WT and ASC KO mice were LPS-primed (1 *μ*g/ml, 2 h) and then treated with TPEN (10 *μ*M, 4 h) with IL-1*β* release measured by ELISA (i), and cell death by LDH release (ii). All data are presented as the mean±S.D. from at least four separate experiments. ***P<0.001, **P<0.01, *P<0.05

Caspase-1 activation is regulated by multi-protein complexes called inflammasomes. The NLRP3 inflammasome is generally regarded as a sensor of sterile injury, and given that TPEN is a sterile stimulus we hypothesised that TPEN-induced IL-1*β* release occurred through activation of the NLRP3 inflammasome. We reported recently that PP1/PP2A phosphatase inhibitors such as calyculin A (CA) and okadaic acid are potent inhibitors of multiple inflammasomes.^23^ Incubation of LPS-primed primary peritoneal macrophages with 10 or 50 nM CA completely inhibited TPEN-induced IL-1*β* processing and release (Figure 4a), consistent with our previous observations on inflammasome inhibition.^23^ To test the involvement of NLRP3, we incubated LPS-primed peritoneal macrophages with the NLRP3 inflammasome inhibitor glyburide,^24^ which also significantly inhibited TPEN-induced IL-1*β* release (Figure 4b). When treated with TPEN, macrophages from NLRP3 KO mice secreted significantly less IL-1*β* compared with WT macrophages, although cell death responses were not affected (Figure 4c). Likewise, macrophages isolated from ASC KO mice also secreted significantly less IL-1*β* than WT in response to TPEN (Figure 4d). Together, these data strongly suggest that Zn^2+^ depletion activates the processing and secretion of IL-1*β,* which is at least partially via a NLRP3-inflammasome/caspase-1-dependent pathway.

**Figure 5:**
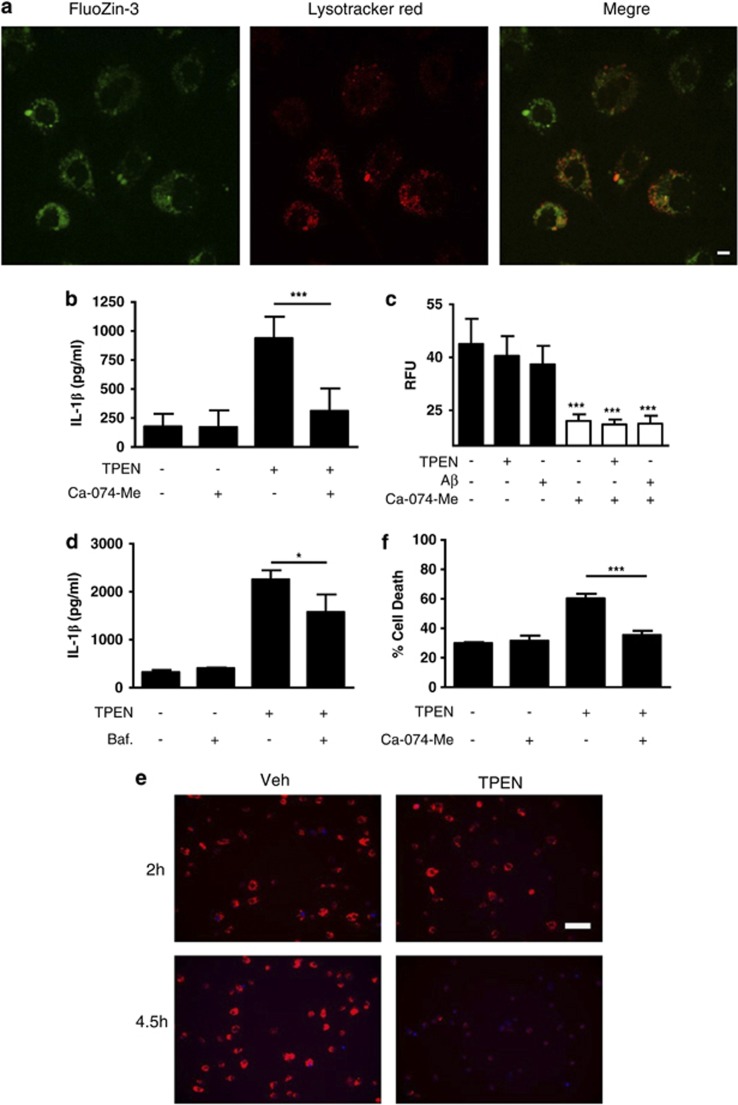
TPEN-induced NLRP3 inflammasome activation depends upon a loss of lysosomal integrity. (**a**) LPS-primed (1 *μ*g/ml, 2 h) peritoneal macrophages were loaded with FluoZin-3 and Lysotracker Red and imaged using a spinning disc confocal microscope. Scale bar represents 5 *μ*m. (**b**) LPS-primed peritoneal macrophages were incubated with the cathepsin B inhibitor Ca-074-Me (80 *μ*M) before incubation with TPEN (10 *μ*M, 4 h) with IL-1*β* release measured by ELISA. (**c**) LPS-primed peritoneal macrophages were incubated with TPEN (10 *μ*M, 4 h) or A*β* (5 *μ*M, 4 h) after which cells were lysed in hypotonic lysis buffer. Cathepsin B/L-dependent cleavage of the fluorogenic substrate Z-Phe-Arg-AMC (40 *μ*M) was measured by an increase in fluorescence (excitation 335 nm, emission 460 nm). The cathepsin B inhibitor Ca-074-Me (100 *μ*M) was included as a control. (**d**) LPS-primed peritoneal macrophages were incubated with bafilomycin A (100 nM) before incubation with TPEN (10 *μ*M, 4 h) with IL-1*β* release measured by ELISA. (**e**) LPS-primed (1 *μ*g/ml, 2 h) peritoneal macrophages were loaded with Lysotracker Red and then incubated with DMSO (0.5%, Veh), or TPEN (10 *μ*M) for 2 and 4.5 h. Snap shot images of live cells were taken using a BD Pathway Bioimager. Hoechst was included in the culture media allowing labelling of cells in which plasma membrane integrity was compromised. Scale bar=50 *μ*m. (**f**) LPS-primed peritoneal macrophages were incubated with TPEN (10 *μ*M, 6 h) plus and minus Ca-074-Me (100 *μ*M) with cell death measured by release of LDH. All data are presented as the mean±S.D. from at least four separate experiments. All images are representative. ***P<0.001, *P<0.05

Canonical NLRP3 inflammasome activation is reported to depend upon one, or a combination of signals that include K^+^ ion efflux,^25^ reactive oxygen species (ROS) generation,^26^ or destabilisation of lysosomal membranes.^27^ Destabilisation of lysosomal membranes and the release of cathepsins from the lysosome into the cytosol is reported to activate the NLRP3 inflammasome in response to DAMPs such as Alum and amyloid-beta (A*β*) and infection by certain pathogens (e.g., *Listeria monocytogenes*).^27, 28, 29^ Recently, K^+^ efflux was linked to NLRP3 activation in response to DAMPs and reagents that also damage lysosomal membranes, perhaps identifying it as a general mechanism regulating inflammasome activation.^30^ We tested whether TPEN treatment of LPS-treated macrophages induced a ROS response. However, using the ROS indicator MitoSOX TPEN failed to induce a detectable increase in ROS, whereas sphingosine, a recently characterised activator of NLRP3,^23^ induced a robust increase (data not shown). Labile Zn^2+^, as detected with the fluorescence probe FluoZin-3, localises to lysosomes in T cells^31^ and in cultured neurones.^32^ We identified partial co-localisation between FluoZin-3 and the lysosome-specific dye lysotracker red in LPS-treated peritoneal macrophages (Figure 5a). Therefore, we tested whether TPEN-induced NLRP3 inflammasome activation and IL-1*β* secretion were dependent upon lysosomal destabilisation. First, we showed that incubation of primary peritoneal macrophages with the cathepsin B inhibitor Ca-074-Me (80 *μ*M) resulted in an inhibition of TPEN-induced IL-1*β* release (Figure 5b), suggesting that TPEN influenced lysosomal membrane stability. Furthermore, pre-treatment of LPS-primed macrophages with the vacuolar H1-ATPase inhibitor bafilomycin A caused a partial but significant inhibition of TPEN-induced IL-1*β* release, further suggesting that a loss of lysosomal integrity is important (Figure 5d). Neither TPEN nor A*β* (previously reported to activate NLRP3 via lysosomal destabilisation^28^) increased cathepsin activity in peritoneal macrophage cell lysates (Figure 5c), further suggesting that lysosomal membrane disruption and a redistribution of lysosomal contents are required for TPEN-induced IL-1*β* release. We loaded peritoneal macrophages with Lysotracker Red and then incubated them with DMSO (0.5%, Veh) or TPEN (10 *μ*M) for up to 4.5 h. We then took ‘snap-shot' images of the live cells using a BD Pathway Bioimager. The fluorescent DNA dye Hoechst was included in the culture media allowing labelling of cells in which plasma membrane integrity was compromised. Loss of Lysotracker red labelling correlated with an increase in Hoechst labelling in the TPEN-treated cells (Figure 5e). Lysosomal membrane permeabilisation is a well-characterised mechanism of cell death where release of cytotoxic cathepsins into the cytosol reduces cell viability.^33^ To further establish that Zn^2+^ depletion was inducing this effect, we investigated whether the cathepsin B inhibitor Ca-074-Me reduced TPEN-induced cell death. LPS-primed peritoneal macrophages were incubated with TPEN (10 *μ*M) for the slightly longer time of 6 h (to induce a robust cell death response) plus and minus Ca-074-Me (100 *μ*M) with cell death measured by release of lactate dehydrogenase (LDH). Ca-074-Me was protective against TPEN-induced cell death (Figure 5f). Thus, from these data we conclude that Zn^2+^ depletion induces inflammasome activation and cell death via a disruption of lysosomal integrity. Even though both caspases 1 and 8 were activated by TPEN, neither a caspase-8 nor a caspase-1 inhibitor reduced TPEN-induced cell death (Figure 3). NLRP3 KO macrophages also released comparable levels of LDH compared with WT macrophages in response to TPEN (Figure 4). These data suggest that pyroptosis is probably not the mechanism of cell death we observe, and the rapid onset of membrane permeabilisation (4–6 h) suggests that the death is not apoptotic. Our data suggest that the mechanism of TPEN-induced cell death is lysosomal in nature. Lysosomal damage is known to contribute to multiple cell death paradigms,^33^ and lysosomal protease-dependent cell death, in the presence of active caspases, has been reported previously (e.g., see Di Piazza *et al.*^34^ and Nylandsted *et al.*^35^).

The molecular sensors for Zn^2+^ in this system are unknown. Approximately 2800 proteins are known to bind Zn^2+^,^7^ and the effect we observe here could be due to either a direct or an indirect effect of the function, or loss of function, of one or many of these proteins. Given the importance of intracellular Zn^2+^, it is expected that a drop in its levels would elicit a prototypical response to danger; namely the secretion of IL-1*β*. There is also literature reporting the direct stabilising effects of Zn^2+^ on biological membranes, and in particular lysosomal membranes.^36, 37^ Thus, the effects of Zn^2+^ depletion on lysosomal membranes could depend upon multiple factors.

Given its prevalence and association with inflammation and disease, Zn^2+^ deficiency potentially represents a major co-morbidity for disease. Rodents fed Zn^2+^-deficient diets suffer exacerbated pathology in models of colitis^38^ and polymicrobial sepsis.^39^ Humans with Alzheimer's disease (AD) are also Zn^2+^ deficient.^40, 41^ In this respect, AD is particularly interesting as we know that aggregated A*β*, a pathological feature of AD, induces NLRP3 inflammasome activation via a mechanism dependent upon destabilisation of lysosomes and cathepsin B.^28^ The memory deficits that occur in the APP/PS1 mouse model of AD also appear to be entirely dependent upon the NLRP3 inflammasome.^42^ In a negative feedback loop, Zn^2+^ inhibits NF-κB-dependent inflammation via the Zn^2+^ transporter ZIP8, itself regulated by NF-κB, and does this via an inhibition of IκB kinase.^43^ Under conditions of Zn^2+^ deficiency Liu *et al.*^43^ report excessive inflammation in a model of sepsis as a result of losing this negative feedback. Thus, in addition to the mechanism reported by Liu *et al.*,^43^ we have identified that activation of the NLRP3 inflammasome can also contribute to inflammation under conditions of Zn^2+^ deficiency, and that this is likely via a direct effect on lysosomal membrane integrity. These data therefore suggest that Zn^2+^ deficiency could be a contributor to disease in which there is an IL-1*β*-dependent inflammatory response. Zn^2+^ deficiency is easily resolved using dietary Zn^2+^ supplements,^13, 14^ and this could potentially represent a simple treatment for inflammatory disease where plasma Zn^2+^ levels are found to be low.

## Materials and Methods

### Materials

RPMI 1640 and DMEM culture media, fetal bovine serum, glutamine, and a streptomycin/penicillin antibiotic solution were all purchased from Invitrogen (Paisley, UK). Bacterial LPS (*Escherisha coli* 026:B6), ZnPyr (zinc salt), TTM, TPEN, DTPA, pyrithione, CA, glyburide, and bafilomycin were purchased from Sigma (Gillingham, UK). Ac-YVAD-CHO, IETD-CHO, and Ca-074-Me were purchased from Merck Chemicals Ltd (Darmstadt, Germany). Salicylaldehyde isonicotinoyl hydrazone (SIH) was purchased from ChemBridge (San Diego, CA, USA). FluoZin-3 and Lysotracker Red were purchased from Invitrogen. The anti-mouse IL-1*β* antibody was from R&D Systems (Abingdon, UK). The XIAP and caspase-8 antibodies were purchased from Cell Signalling (Danvers, MA, USA), the actin antibody was from Sigma, and the caspase-1 antibody was from Santa Cruz Biotechnology (Dallas, TX, USA). A*β* peptide (1-42) was purchased from rPeptide (Bogart, GA, USA).

### Mice

NLRP3 and ASC KO mice were generously provided by Dr. Vishva Dixit, Genentech.^44, 45^ C57BL/6J mice were purchased from Harlan (Blackthorn, UK).

### *In vivo* sepsis

All procedures on animals conformed to the requirements of the UK Animal (Scientific Procedures) Act, 1986. Male C57BL/6 mice (25–30 *g* bodyweight) were injected i.p. with a septic dose of LPS (*E. coli* 0127:B8, Sigma, 5 mg/kg) prepared in sterile 0.9% saline. 1 h after LPS injection the mice received a second injection that was vehicle (10% DMSO in saline), 1 mg/kg TPEN, or 10 mg/kg TPEN. 3 h after the second injection the animals were terminally anaesthetised with 3% isoflurane, lavage fluid was collected, and blood collected via cardiac puncture. Samples were centrifuged and supernatants collected and frozen until analysis.

### Cell culture

Primary peritoneal macrophages were prepared from adult, male C57BL/6 mice, as described previously.^46^ Peritoneal macrophages were cultured at a density of 5 × 10^5^ cells/well in RPMI 1640 media supplemented with 5% FCS, 100 U/ml penicillin and 100 *μ*g/ml streptomycin. 24 h after culture cells were treated with LPS (1 *μ*g/ml, 2 h) before a 4 h incubation with 10 *μ*M TPEN (or other treatments where indicated). Supernatants and lysates were harvested for subsequent analyses. For the imaging experiments, primary peritoneal macrophages or J774 macrophages were treated with LPS (1 *μ*g/ml, 2/4 h) and then loaded with FluoZin-3 and Lysotracker Red (see below).

### Imaging experiments

#### FluoZin-3 and Lysotracker Red imaging

LPS-treated J774 macrophages were incubated with 10 *μ*M FluoZin-3 ester plus 0.02% pluronic acid and 100 nM Lysotracker Red for 30 min. After incubation, cells were washed three times with media to remove excess dye and were then imaged.

Co-localisation of FluoZin-3 and Lysotracker Red was observed using a Zeiss Axio-Observer Z1 microscope equipped with a CSU-X1 spinning disc confocal microscope (Yokagowa, Tokyo, Japan) and imaged using an Evolve EM CCD camera (Photometrics, Tucson, AZ, USA). Live cell kinetics of Zn^2+^ loading was performed using a BD Pathway Bioimager with liquid handling (see below). All offline analysis of images and movies used ImageJ software (http://rsb.info.nih.gov/ij/). In ImageJ, cells were selected as regions of interest (ROI) and at least 15 cells per field of view were analysed. The fluorescence (F) data are expressed as a fluorescence change relative to the initial baseline fluorescence value (Fo).

#### Lysotracker Red and Hoechst imaging

LPS-treated peritoneal macrophages (2 h, 1 *μ*g/ml) were incubated with Lysotracker Red (100 nM) for 30 min. Cells were washed three times and incubated with media containing 2 *μ*g/ml Hoechst plus either vehicle (0.5% DMSO) or TPEN (10 *μ*M). Cells were then imaged at regular intervals up to 4.5 h. For longer incubations (>2 h), cells were reloaded with Lysotracker for 30 min before imaging. Images were acquired on a Pathway Bioimager 855 (BD, Oxford, UK) with laser autofocus using a × 20/0.75 Olympus objective (Southend, UK) and the following filtre setups: FluoZin-3 Ex. 334/10 Di. FURA/FITC Em. 520; Rhodamine Ex. 555/28 Di. FURA/FITC Em. 645/75; Hoechst Ex. 380/10 Di. FURA/TRITC Em. 520. For snap shot images, settings were determined manually. The BD Pathway and the spinning disc confocal microscopes are part of the Core Bioimaging Facility at the Faculty of Life Sciences, University of Manchester (http://www.ls.manchester.ac.uk/research/facilities/bioimaging/).

### Western blotting

Supernatants and lysates were harvested and prepared in sample buffer containing 1% *β*-mercaptoethanol. Samples were boiled and then electrophoresed on 12% SDS-acrylamide gels. For the caspase-1 blots, supernatants were concentrated using Amicon Ultra centrifugal filtres (Millipore, Darmstadt, Germany). Proteins were transferred to a nitrocellulose membrane and blotted with primary antibodies, followed by HRP-conjugated secondary antibodies, and subsequent exposure using enhanced chemi-luminesence reagents (ECL, Amersham, UK).

### Detection of IL-1*β* and IL-6 by ELISA

Measurement of IL-1*β* released into macrophage culture supernatants and peritoneal lavage fluid, and measurement of IL-6 in lavage fluid, was done using specific mouse IL-1*β* and IL-6 ELISA kits (R&D Systems) following manufacturer's instructions.

### Cathepsin B/L assay

Cells were lysed in hypotonic lysis buffer (25 mM HEPES, 5 mM EGTA, 5 mM DTT, pH 7.5) on ice, and lysates were mixed with 2 × reaction buffer (0.2 M sodium acetate buffer, 4 mM EDTA, 4 mM DTT, pH5.5). Cathepsin B/L-dependent cleavage of the fluorogenic substrate Z-Phe-Arg-AMC (40 *μ*M) was measured by an increase in fluorescence (excitation 335 nm, emission 460 nm). Data are expressed as relative fluorescence units.

### Lactate dehydrogenase assay

Cell death was recorded by measuring the release of the enzyme LDH from the cells using the CytoTox-96 assay (Promega, Southampton, UK) according to manufacturer's instructions.

### Data analysis

Cell culture data are presented as the mean±S.D. of at least three separate cultures. Groups of data were analysed by one-way ANOVA followed by Bonferroni's multiple comparison test. Statistical significance was assumed when *P*<0.05. All western blots presented are representative of three independent experiments from three separate cultures. The *in vivo* experiment used six animals per group.

## Abbreviations

A*β*: Amyloid beta
ASC: apoptosis-associated speck-like protein containing a caspase recruitment domain
CA: calyculin A
DAMPs: damage-associated molecular patterns
DMSO: dimethyl sulfoxide
DTPA: diethylenetriaminepentaacetic acid
IL: interleukin
LPS: lipopolysaccharide
LDH: lactate dehydrogenase
NLRP3: NACHT, LRR, and PYD domains-containing protein 3
PRRs: pattern recognition receptors
PYD: pyrin domain
ROS: reactive oxygen species
SIH: salicylaldehyde isonicotinoyl hydrazone
TPEN: *N,N,N',N'*-tetrakis-(2-pyridylmethyl) ethylenediamine
XIAP: X-linked inhibitor of apoptosis protein

## Notes

The authors decalre no conflict of interest.
